# Identification of the Complete Mitochondrial Genome of the Malayan Pangolin (*Manis javanica* Demarest, 1822) and Its Evolutionary Relationship with Other Pangolin Species

**DOI:** 10.3390/genes17050498

**Published:** 2026-04-23

**Authors:** Xiaobing Guo, Shanghua Xu, Wenhui Liang, Miaomiao Jia, Yong Pan, Yuan Lin, Xinyue Li

**Affiliations:** 1 Guangxi Key Laboratory of Special Non-Wood Forests Cultivation and Utilization, Guangxi Forestry Research Institute, Nanning 530002, China; 2 Guangxi Forestry Laboratory, Nanning 530002, China

**Keywords:** *Manis javanica*, mitochondrial genome, phylogenetic analysis, non-invasive sampling

## Abstract

Background: Pangolins are critically endangered mammals, and a comprehensive understanding of their genetic diversity is crucial for effective conservation. The mitochondrial genome serves as a vital molecular marker for phylogenetic and population genetic studies. Obtaining genetic material from these elusive animals non-invasively remains a challenge. This study aimed to sequence and characterize the complete mitochondrial genome of *Manis javanica* and explore the phylogenetic relationships among pangolin species. Methods: The complete mitochondrial genome was sequenced from a saliva-derived sample. Standard procedures for DNA extraction, amplification, and sequencing were employed. The genome was assembled and annotated using bioinformatic tools. Phylogenetic analysis was conducted based on the cytochrome c oxidase subunit I (COXI) gene sequences from nine pangolin species, with the resulting tree constructed using the maximum-likelihood method. Results: The complete mitochondrial genome of *M. javanica* (GenBank accession: PP110760) is a circular molecule of 16,573 bp, containing 13 protein-coding genes, 22 tRNA genes, 2 rRNA genes, and a control region. The overall base composition showed a lower GC content (43.83%) than AT content (56.17%). Phylogenetic analysis based on COXI sequences delineated the nine species into three distinct genera: *Manis*, *Phataginus*, and *Smutsia*. Within the genus *Manis*, *Manis pentadactyla* was identified as the closest relative to *M. javanica*. The newly described species *Manis mysteria* was found to be closer to *Manis culionensis* and *Manis crassicaudata* than to other congeners. Furthermore, the analysis indicated that African pangolins diverged earlier than Asian pangolins. Conclusions: This study successfully demonstrates the feasibility of extracting and sequencing the complete mitochondrial genome from saliva samples, providing a valuable non-invasive method for future genetic studies on pangolins. The genomic data and phylogenetic results offer significant molecular insights that will benefit the genetic management and conservation of critically endangered pangolin resources.

## 1. Introduction

**Figure 1:**
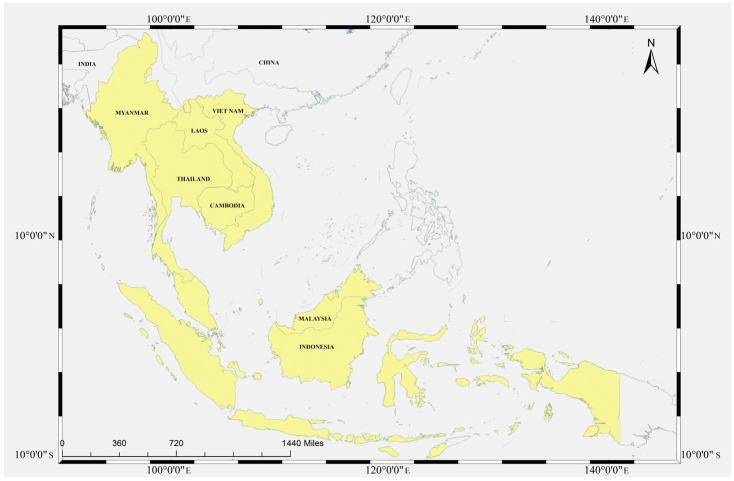
Distribution range of *Manis javanicain* Southeast Asia and southern China.

This study focuses on the Malayan pangolin (*Manis javanica*), a myrmecophagous mammal whose contemporary distribution spans mainland and insular Southeast Asia—including Myanmar, Thailand, Laos, Cambodia, Vietnam, Peninsular Malaysia, Singapore, Sumatra, Borneo, and Java—with its northern range extending into Yunnan provinces of southern China [1,2]. Here it overlaps ecologically with the Chinese pangolin (*Manis pentadactyla*) along montane transition zones [3]. Listed as Endangered on the IUCN Red List and included in CITES Appendix I since 2016, *M. javanica* faces persistent pressure from illegal harvest and habitat modification across its range [4,5]. Its biogeographic context within Asia is illustrated in Figure 1.

Globally, the order Pholidota comprises eight extant species partitioned into African (*Phataginus tetradactyla*, *Phataginus. tricuspis*, *Smutsia gigantea*, *Smutsia. temminckii*) and Asian (*Manis crassicaudata*, *Manis culionensis*, *M. javanica*, *M. pentadactyla*) genera [6]. All species are scale-bearing, obligate ant/termite feeders that contribute significantly to ecosystem regulation [7]. Their collective distributions are summarized in Supplementary Figure S1.

Historically abundant across their ranges, Asian pangolins have experienced severe population decline in recent decades due to intensive poaching for scales and meat, exacerbated by landscape fragmentation [8]. More than one million individuals are estimated to have been trafficked globally since 2000, with primary market destinations in China and Vietnam [9]. Strengthened legal protections in the China and CITES Appendix I listing reflect elevated conservation prioritization. Captive rescue and breeding initiatives have emerged as critical components of species recovery, requiring accurate genetic data to manage population viability and preserve adaptive diversity [10,11,12].

Against this backdrop, current pangolin genetic monitoring typically relies on DNA from tissue, scale, or blood samples—sources demanding invasive or terminal collection that is poorly aligned with field-based conservation studies [13,14]. Non-invasive fecal DNA, while operationally convenient, commonly yields degraded templates complicated by PCR inhibitors and high microbial load, limiting reliable mitochondrial genome assembly [15,16,17]. Saliva sampling via buccal swabs represents a promising intermediate approach that is less invasive than biopsies but that generally provides superior eukaryotic DNA integrity compared to scat [18,19]. Although buccal swab protocols are established in human forensics and select wildlife applications, standardized saliva-based workflows tailored to pangolin genomics and specifically whole mitogenome sequencing remain underdeveloped.

Furthermore, existing pangolin phylogenetic frameworks depend heavily on mitogenomes derived from confiscated or archival specimens, restricting fine-scale spatiotemporal inference. Fresh, wild-sourced mtDNA acquired through non-invasive methods could substantially improve haplotype resolution but remains underrepresented. Regionally variable molecular markers (e.g., COX1 substitutions) may additionally illuminate local adaptation or metapopulation structure, though validation against high-fidelity references is required.

To address these gaps, this study aims to achieve the following: (1) establish a field-applicable workflow for recovering complete mitochondrial genomes from pangolin saliva using commercial magnetic bead kits and NGS; (2) characterize the mtDNA architecture of *M. javanica* from Guangxi, comparing structural features with Malaysian conspecifics to identify conserved and polymorphic regions; and (3) evaluate COX1 nucleotide and amino acid variability as a potential indicator of regional differentiation. By validating saliva as a practical mtDNA source, we seek to deliver a scalable, welfare-compatible genetic tool for conservation practitioners while contributing reference data to support pangolin reintroduction planning.

## 2. Materials and Methods

**Figure 2:**
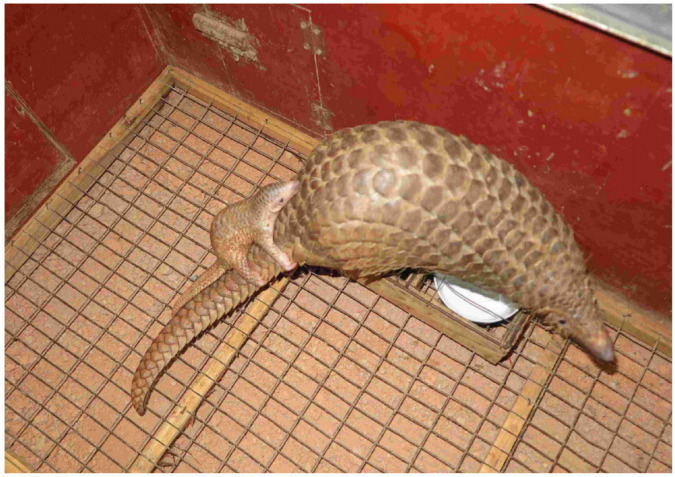
Photography of *M. javanica* taken by Xiaobing Guo in August 2025.

The pangolins used in this study were obtained from the Pangolin Rescue and Breeding Center of the Guangxi Forestry Research Institute, Nanning, Guangxi Zhuang Autonomous Region, People’s Republic of China (108.3458 E, 22.9393 N). The specimen was deposited at the Guangxi Key Laboratory of Special Non-wood Forest Cultivation & Utilization, Nanning, Guangxi Zhuang Autonomous Region, China (contact: Baocai Li, 957065217@qq.com) under voucher number GFRI1103. A species reference image for the species is shown in Figure 2.

Before sampling, the pangolins were secured, and oral swabs and 5 mL sterilized centrifuge tubes were prepared. A buccal swab was dipped in pangolin saliva and quickly transferred to a sterilized centrifuge tube, which was then capped. This process is repeated five times. After successful sampling, the centrifuge tube was stored in a liquid nitrogen tank for later use.

Genomic DNA from a pangolin was extracted via the Magnetic Universal Genomic DNA Kit (Tiangen Biotech Co., Ltd., Beijing, China) following the manufacturer’s protocol. The Illumina NovaSeq 6000 platform (Illumina, Inc., San Diego, CA, USA) was used to sequence the whole genome of *M. javanica*, with a sequencing depth of 8.81×. The complete mitochondrial sequence was assembled via Geneious Primer v.2024.0.5 [20]. The analysis of the mitochondrial genome structure was conducted via the Tutools platform (http://www.cloudtutu.com), a free online data analysis website, and annotated via MITOS 2 [21].

To elucidate the phylogenetic status of *M. javanica* in Pholidota, eight currently available complete mitogenomes of Pholidota obtained from GenBank were used in phylogenetic analyses. Four species of Canivora (*Canis lupus* KU696410, *Arctocephalus pusillus* NC008417, *Acinonyx jubatus* AY463959 and *Crocuta crocuta*, JF894378) and one species of Artiodactyla (*Alces alces* NC020677) were used as outgroups. The phylogenetic tree was reconstructed via the maximum likelihood (ML) method on the basis of COXI sequences via MEGA 11 [22]. The number of bootstrap replicates was set to 1000 with automatic model prediction in the ML analyses.

## 3. Results

### 3.1. Mitochondrial Genome Assembly and Characterization of Manis javanica

**Figure 3:**
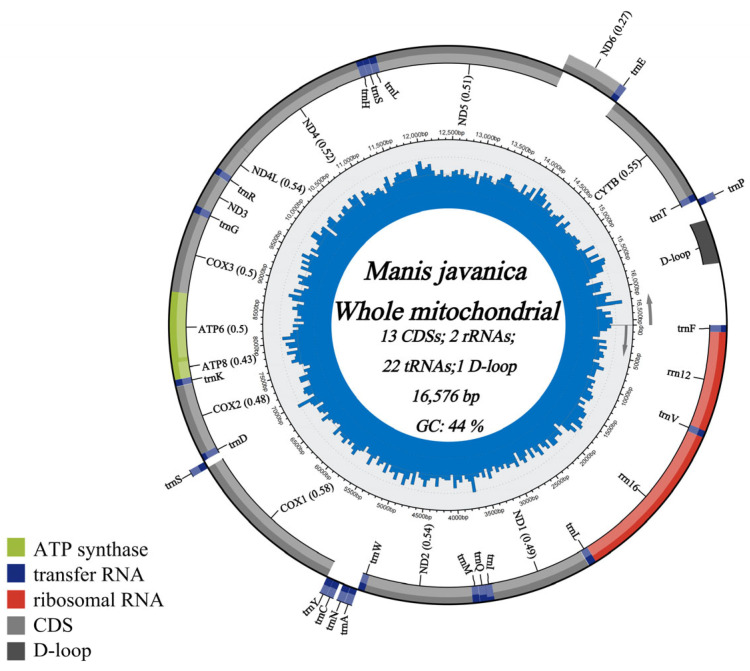
The complete mitochondrial genome of *M. javanica*. The innermost circles depict the GC content, and the outermost circle indicatescircles indicate the arrangements of genes: inner genes from the forward strand, and outer genes from the reverse strand, with CDSs in gray, rRNAs in red, ATP synthase in green and tRNAs in purple.

In this study, we successfully characterized the complete mitochondrial genome of the critically endangered *M. javanica*, which has been deposited in GenBank under accession number PP110760. The newly sequenced mitochondrial genome is 16,576 bp in length and exhibits a structure typical of vertebrates, comprising 13 protein-coding genes (PCGs), 22 transfer RNA (tRNA) genes, 2 ribosomal RNA (rRNA) genes, and 1 control region (D-loop) (Figure 3), similar to typical vertebrate mitochondrial DNA [23,24,25,26,27]. 

Among the PCGs are 7 NADH dehydrogenase subunits (ND1–6, ND4L), 3 cytochrome c oxidase subunits (COXI–III), 2 ATP synthase subunits (ATP6 and ATP8), and cytochrome b(Cytb). The ND6 gene and seven tRNA genes (tRNA-Ala, -Asn, -Cys, -Tyr, -Ser(UCN), -Glu, and -Pro) are encoded on the light strand, while all other genes are encoded on the heavy strand. The D-loop is the only major non-coding region. The longest PCG is ND5 (1821 bp), and the shortest is ATP8 (204 bp). Regarding start codons, ND6 uses ATA, ND2 and ND3 use ATT, ND5 uses GTG, and the remaining nine PCGs use ATG. The stop codons for COXII, ATP6, ATP8, ND4L, and ND5 are TAA, while Cytband COXI use AGA. The genes ND1, ND2, ND3, ND4, ND6, and COXIII possess incomplete stop codons (T or TA) (Table 1).

### 3.2. Comparative Analysis of COX1 Sequences Between Malaysian and Guangxi Populations

A comparison of the COX1 nucleotide sequences from *M. javanica* individuals from Malaysia and Guangxi, China, revealed a high degree of conservation, with 99% sequence identity. Analysis identified 43 variable sites, of which 42 were synonymous substitutions and one was a nonsynonymous substitution (Table S1). This single nonsynonymous mutation results in an amino acid change at position 155: isoleucine (I) in the Malaysian individual was replaced by valine (V) in the Guangxi individual (Table S2).

### 3.3. Phylogenetic Analysis Based on COXI Sequences

**Figure 4:**
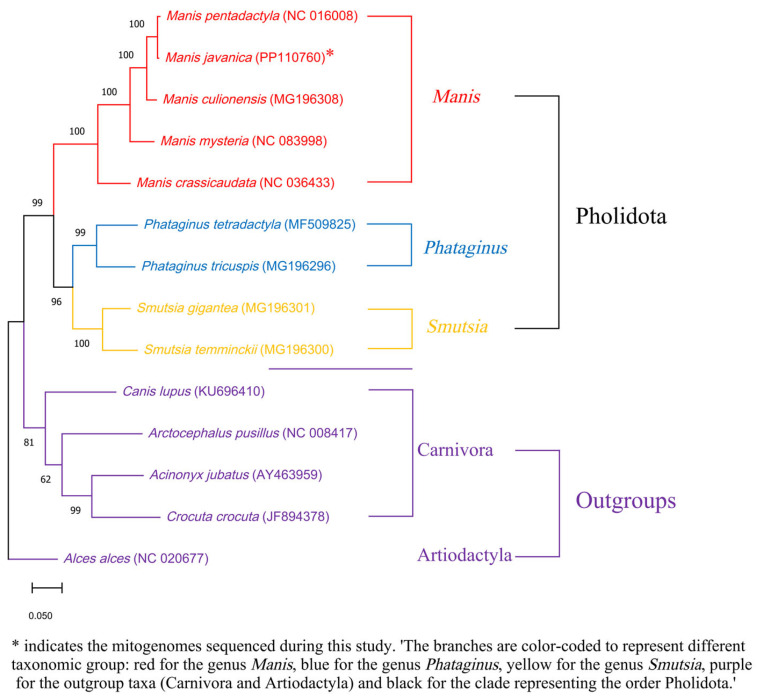
Maximum likelihood tree for Malayan pangolins and 8 other pangolin species constructed based on COXI sequences using MEGA 11.

Phylogenetic reconstruction using COXI sequences from nine pangolin species resolved them into three distinct genera: *Manis*, *Phataginus*, and *Smutsia*. Within the genus *Manis*, *M. pentadactyla* was identified as the closest relative to *M. javanica*. The newly described species *M. mysteria* [28] formed a clade with *M. culionensis* and *M. crassicaudata*. Furthermore, the topology indicated that the African pangolin genera (*Phataginus* and *Smutsia*) diverged earlier than the Asian pangolins (*Manis*), providing a temporal framework for pangolin evolution (Figure 4). 

The maximum likelihood tree was constructed using MEGA 11, with bootstrap values supporting the nodes. Outgroups included *Alces alces*, *Acinonyx jubatus*, *Crocuta crocuta*, *Canis lupus*, and *Arctocephalus pusillus*.

The number under the internode represents the bootstrap value. The GenBank accession numbers used are listed in brackets after the species names. The scale bar indicates the unit length of the value of the difference between sequences. The following sequences were used: NC_016008 [29], MG_196308 [30], NC_083998 [28], NC_036433 [30], MF_509825 [31], MG_196296, MG_196301, MG_196300 [30], KU_696410 [32], NC_008417 [33], AY_463959 [34], JF_894378 [35], and NC_020677 [36].

## 4. Discussion

The mitochondrial genome of *M. javanica* presented in this study provides insights into the genetic structure and phylogenetic relationships of pangolins. The circular mitochondrial genome’s unique composition of coding sequences, tRNA genes, rRNA genes, and control regions reflects the conserved mitochondrial DNA structure among vertebrates [37]. The observed nucleotide content, with a greater proportion of AT than GC, is characteristic of mitochondrial genomes and might influence transcription and replication processes within the mitochondrial compartment [38].

The feasibility of extracting the genome from saliva samples and amplifying the mitochondrial genome is demonstrated, opening new avenues for genetic diversity research on pangolins. This noninvasive sampling method could be particularly useful for monitoring pangolin populations in their natural habitats where invasive sampling techniques may not be feasible. A comparison of COX1 nucleotide sequences from *M. javanica* samples from Malaysia and Guangxi revealed high consistency, validating the use of COX1 markers for analyzing the genetic diversity of this species. However, amino acid sequence comparison revealed that the 155th amino acid was isoleucine (I) in the Malaysia sample and valine (V) in the Guangxi sample. This variation provides a potential molecular marker for distinguishing regional populations. As valine and isoleucine are essential amino acids obtained through the diet [39], the observed substitution in the Guangxi population may be influenced by long-term dietary factors, though the underlying mechanisms warrant further exploration.

Phylogenetic analysis based on COXI sequences underscores the close relationships among pangolin species. The clustering of pangolins into three genera—*Manis*, *Phataginus*, and *Smutsia*—provides a robust taxonomic framework for future studies. The close relationship between *M. pentadactyla* and *M. javanica* suggests a shared evolutionary history, which should inform cross-species conservation strategies. The placement of the newly described species *M. mysteria*, showing closer affinity to *M. culionensis* and *M. crassicaudata*, enhances our understanding of the phylogenetic diversification within the genus *Manis*. Furthermore, the earlier divergence of African pangolins compared to Asian pangolins adds a temporal dimension to their evolutionary history, highlighting the need to consider phylogenetic distinctiveness in conservation prioritization.

Our study has several constraints that merit consideration. First, despite successful mtDNA amplification, salivary DNA fragmentation may limit recovery of full-length nuclear loci, and residual bacterial/feed contamination could affect rare variant calling—future work should validate findings with additional tissue-matched controls [40]. Second, current inferences rely primarily on a single mitochondrial marker (COX1) and limited regional samples; broader geographic sampling combined with whole mitogenome or nuclear SNP datasets would strengthen population structure analyses [41]. Third, while dietary influences are hypothesized to explain the Ile155Val substitution, environmental gradients and local adaptation mechanisms cannot be fully disentangled without metabolomic or transcriptomic follow-up [42]. Future efforts should prioritize expanding noninvasive sampling across Southeast Asian pangolin ranges, integrating nuclear gene panels to verify phylogeographic patterns, and applying target capture methods to overcome saliva DNA degradation in degraded-field conditions.

## 5. Conclusions

This study successfully sequenced and assembled the complete mitochondrial genome of *M. javanica* from a minimally invasive saliva-derived DNA sample, demonstrating the viability of buccal swabbing as a practical starting material for conservation genomics. The workflow efficiently recovered sufficient template for long-range PCR and NGS, overcoming common hurdles of degraded field samples. Beyond the assembly of the circular mtDNA structure, comparative analysis identified a notable amino acid variation (Ile155Val in COX1) between Guangxi and Malaysian populations, proposing a candidate marker for regional traceability relevant to wildlife forensics. Furthermore, phylogenetic reconstruction based on COXI sequences elucidated interspecific relationships with *Manis* and reaffirmed the deep evolutionary split between African and Asian pangolin clades. Collectively, the genomic resource and the validated non-invasive sampling strategy significantly expand the molecular toolkit available for genetic monitoring, origin verification, and informed management of critically endangered pangolin populations.

## Figures and Tables

**Table 1:** Annotation of the mitochondrial genome of *M. javanica*.

Gene Region	Abbreviation	Start Position	Stop Position	Start Codon	Stop Codon	Anticodon	Size (bp)	Strand
tRNA-Phe	trnF	1	68			GAA	68	+
12S rRNA	rrnS	69	1028				960	+
tRNA-Val	trnV	1029	1094			TAC	66	+
16S rRNA	rrnL	1095	2665				1571	+
tRNA-Leu2	trnL2	2666	2739			TAA	74	+
ND1	nad1	2743	3698	ATG	TAA		956	+
tRNA-Ile	trnI	3699	3767			GAT	69	+
tRNA-Gln	trnQ	3765	3837			TTG	73	+
tRNA-Met	trnM	3839	3907			CAT	69	+
ND2	nad2	3908	4946	ATA	TAA		1039	+
tRNA-Trp	trnW	4947	5013			TCA	67	+
tRNA-Ala	trnA	5017	5085			TGC	69	-
tRNA-Asn	trnN	5087	5159			GTT	73	-
tRNA-Cys	trnC	5193	5273			GCA	81	-
tRNA-Tyr	trnY	5258	5324			GTA	67	-
COX1	cox1	5326	6876	ATG	AGA		1551	+
tRNA-Ser2	trnS2	6871	6942			TGA	72	-
tRNA-Asp	trnD	6948	7013			GTC	66	+
COX2	cox2	7015	7698	ATG	TAA		684	+
tRNA-Lys	trnK	7701	7764			TTT	64	+
ATP8	atp8	7766	7969	ATG	TAA		204	+
ATP6	atp6	7927	8607	ATG	TAA		681	+
COX3	cox3	8607	9390	ATG	TAA		784	+
tRNA-Gly	trnG	9391	9459			TCC	69	+
ND3	nad3	9460	9805	ATA	TAA		346	+
tRNA-Arg	trnR	9807	9873			TCG	67	+
ND4L	nad4l	9874	10,170	ATG	TAA		297	+
ND4	nad4	10,164	11,541	ATG	TAA		1378	+
tRNA-His	trnH	11,542	11,609			GTG	68	+
tRNA-Ser1	trnS1	11,610	11,669			GCT	60	+
tRNA-Leu1	trnL1	11,670	11,740			TAG	71	+
ND5	nad5	11,741	13,561	ATT	TAA		1821	+
ND6	nad6	13,545	14,069	TCT	TAA		525	-
tRNA-Glu	trnE	14,070	14,138			TTC	69	-
Cytb	cob	14,142	15,276	ATG	TAA		1135	+
tRNA-Thr	trnT	15,282	15,348			TGT	67	+
tRNA-Pro	trnP	15,347	15,408			TGG	62	-
Control region	D-loop	15,575	16,012				438	+

## Data Availability

The genome sequence data that support the findings of this study are openly available in the NCBI GenBank [https://www.ncbi.nlm.nih.gov] (https://www.ncbi.nlm.nih.gov/nuccore/PP110760.1/, accessed on 17 March 2026) under accession no. PP110760.1. The associated BioProject, Biosample, and SRA numbers are PRJNA1091841, SAMN40613622 and SRR28482087, respectively.

## Supplementary Materials

The following supporting information can be downloaded at: https://www.mdpi.com/article/10.3390/genes17050498/s1, Table S1: Nucleotide distribution of the COX1 sequence in two *M. javanica* strains; Table S2: Amino acid distribution of the COX1 sequence in two *M. javanica* strains. Figure S1. Global distribution ranges of all eight extant pangolin species.
