# Supplementary material for: Identification of the Complete Mitochondrial Genome of the Malayan Pangolin (Manis javanica Demarest, 1822) and Its Evolutionary Relationship with Other Pangolin Species
Source: Genes (Basel). 2026 Apr 23;17(5):498. doi: 10.3390/genes17050498 (PMC13205489; doi:10.3390/genes17050498)
Supplement: Supplementary file 1 [file genes-17-00498-s001.zip › Figure S1.pdf]

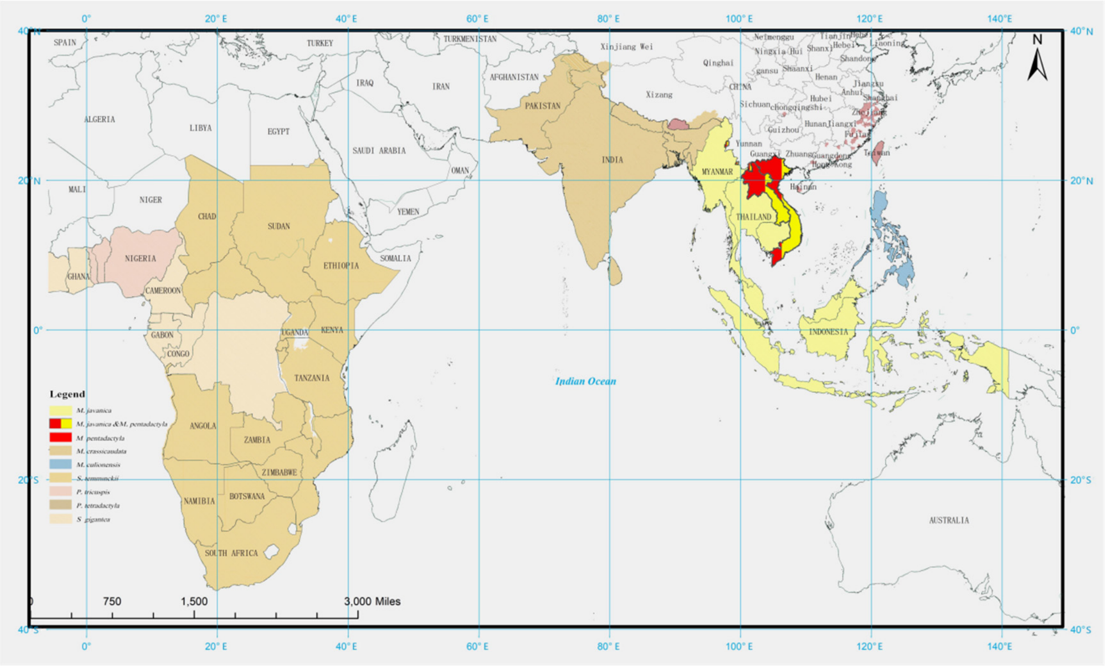

Supplementary Figure S1. Global distribution ranges of all eight extant pangolin species. Data compiled from IUCN Red List spatial datasets
